# In vivo imaging of CNS microglial activation/macrophage infiltration with combined [^18^F]DPA-714-PET and SPIO-MRI in a mouse model of relapsing remitting experimental autoimmune encephalomyelitis

**DOI:** 10.1007/s00259-020-04842-7

**Published:** 2020-05-07

**Authors:** A. R. Coda, S. Anzilotti, F. Boscia, A. Greco, M. Panico, S. Gargiulo, M. Gramanzini, A. Zannetti, S. Albanese, G. Pignataro, L. Annunziato, M. Salvatore, A. Brunetti, P. De Berardinis, Mario Quarantelli, G. Palma, Sabina Pappatà

**Affiliations:** 1 Institute of Biostructure and Bioimaging, National Research Council, Via T. De Amicis 95, 80145 Naples, Italy; 2 IRCCS SDN, Via E. Gianturco 113, 80143 Naples, Italy; 3 Division of Pharmacology, Department of Neuroscience, Reproductive and Odontostomatological Sciences, School of Medicine, University “Federico II”, Via S. Pansini 5, 80131 Naples, Italy; 4 Department of Advanced Biomedical Sciences, University “Federico II”, Via S. Pansini 5, 80131 Naples, Italy; 5 Ceinge Biotecnologie Avanzate s. c. a. r. l., Via G. Salvatore 486, 80145 Naples, Italy; 6 Institute of Biochemistry and Cell Biology, National Research Council, Via P. Castellino 111, 80131 Naples, Italy

**Keywords:** Multiple sclerosis, EAE, Mice, TSPO-PET, SPIO-MRI, Neuroinflammation

## Abstract

**Purpose:**

To evaluate the feasibility and sensitivity of multimodality PET/CT and MRI imaging for non-invasive characterization of brain microglial/macrophage activation occurring during the acute phase in a mouse model of relapsing remitting multiple sclerosis (RR-MS) using [^18^F]DPA-714, a selective radioligand for the 18-kDa translocator protein (TSPO), superparamagnetic iron oxide particles (SPIO), and ex vivo immunohistochemistry.

**Methods:**

Experimental autoimmune encephalomyelitis (EAE) was induced in female SJL/J mice by immunization with PLP_139–151_. Seven symptomatic EAE mice and five controls underwent both PET/CT and MRI studies between 11 and 14 days post-immunization. SPIO was injected i.v. in the same animals immediately after [^18^F]DPA-714 and MRI acquisition was performed after 24 h. Regional brain volumes were defined according to a mouse brain atlas on co-registered PET and SPIO-MRI images. [^18^F]DPA-714 standardized uptake value (SUV) ratios (SUVR), with unaffected neocortex as reference, and SPIO fractional volumes (SPIO-Vol) were generated. Both SUVR and SPIO-Vol values were correlated with the clinical score (CS) and among them. Five EAE and four control mice underwent immunohistochemical analysis with the aim of identifying activated microglia/macrophage and TSPO expressions.

**Results:**

SUVR and SPIO-Vol values were significantly increased in EAE compared with controls in the hippocampus (*p* < 0.01; *p* < 0.02, respectively), thalamus (*p* < 0.02; *p* < 0.05, respectively), and cerebellum and brainstem (*p* < 0.02), while only SPIO-Vol was significantly increased in the caudate/putamen (*p* < 0.05). Both SUVR and SPIO-Vol values were positively significantly correlated with CS and among them in the same regions. TSPO/Iba1 and F4/80/Prussian blue staining immunohistochemistry suggests that increased activated microglia/macrophages underlay TSPO expression and SPIO uptake in symptomatic EAE mice.

**Conclusions:**

These preliminary results suggest that both activated microglia and infiltrated macrophages are present in vulnerable brain regions during the acute phase of PLP-EAE and contribute to disease severity. Both [^18^F]DPA-714-PET and SPIO-MRI appear suitable modalities for preclinical study of neuroinflammation in MS mice models.

## Introduction

Multiple sclerosis (MS) is a chronic neuroinflammatory and neurodegenerative disease mainly characterized neuropathologically by inflammation, gliosis, demyelinating plaques, and axonal and/or neuronal loss [1]. Monocyte-derived macrophages and activated central nervous system (CNS)-resident microglia contribute to pathology of MS [2], although their role in neuronal/axonal loss, the main underlying mechanism of permanent clinical disability in MS, is still controversial [1]. The balance between pro-inflammatory and neuroprotective phenotypes of reactive microglia/macrophages seems to be implicated in demyelination, neuronal damage and tissue repair processes [3]. New PET radioligands and MRI contrast agents targeting molecular and cellular mediators of neuroinflammation represent powerful imaging tools for improving our knowledge on disease mechanisms, progression, and therapy [4, 5]. PET using selective radioligands for the 18-kDa translocator protein (TSPO) has demonstrated increased activated microglia in apparently normal as well as abnormal brain tissue of MS patients [4]. TSPO, previously known as the peripheral benzodiazepine receptor, is a protein found on the outer mitochondrial membrane primarily involved in the transport of cholesterol and steroid synthesis [4]. In the CNS, TSPO is mainly expressed by activated microglia and, to a lower extent, by astrocytes and other cell types [4, 6] including infiltrating macrophages [6] and endothelial cells [7] depending on the neuronal insult, blood-brain barrier (BBB) integrity, and temporal profile.

MRI using superparamagnetic iron oxide (SPIO) particles or ultra-small superparamagnetic iron oxide (USPIO) nanoparticles has emerged as a tool to visualize monocytic/macrophagic infiltration in CNS in MS, although mainly applied to animal studies [8, 9]. Recently, preclinical PET/CT and/or MRI studies combined with immunohistochemistry have been proposed not only for validation and clinical translation of new PET and MRI methods but also to contribute to a better understanding of pathophysiology and therapy of MS [8, 10].

Experimental autoimmune encephalomyelitis (EAE) represents the most widely applied and well-established animal model in MS research that can reproduce clinical and neuropathological aspects of human disease [11]. PET studies in EAE rodent models using [^11^C]PK11195 and/or second-generation TSPO radioligands with higher affinity and specific binding as well as MRI studies using iron oxide particles have demonstrated increased TSPO expression or accumulation of iron oxide particles in the spinal cord and/or brain regions depending of the species, strain, peptide used for immunization [8, 10], and size of particles [8]. To date, few U(SPIO)-MRI and TSPO-PET imaging studies are available in EAE induced by proteolytic protein (PLP) in SJL/J female mice, a model that leads to spinal cord but also widespread cerebral pathology [12, 13] and mimics relapsing remitting MS (RR-MS, the most frequent human MS phenotype). No study has combined the use of both methods to track microglial activation and macrophage infiltration in the same animals and to compare their brain distribution. In this study, we have used high-field 9.4T MR with SPIO and micro PET/CT with [^18^F]DPA-714 to characterize and compare the extent, topography, severity and clinical correlates of TSPO expression and SPIO uptake in the brain of PLP-EAE mice during the acute symptomatic phase. Moreover, we employed light microscopy and confocal double-immunofluorescence studies to characterize changes in TSPO expression and microglial/macrophage activation underlying in vivo signal.

## Material and methods

### Animals and immunization

Thirteen female SJL/J mice 6 weeks old, considered more susceptible to EAE than males [14], were purchased from Charles River (Calco, LC, Italy) and were housed in group cages under standard conditions with free access to food and water. EAE was induced in seven mice by subcutaneous injection (s.i.) on day 0, about 1 week after housing, of a total of 200 μl of an emulsion containing 100 μl of myelin proteolipid protein residues 139–151 HSLGKWLGHPDKF (1 μg/μl PLP139–151—Inbios) and 100 μl of complete Freund’s adjuvant (CFA, Becton Dickinson Italia) at both flanks. The initial immunization was followed by intraperitoneal (i.p.) administration of 100 μl (2 ng/μl in phosphate buffered saline) of Bordetella pertussis toxin (BPX, Sigma-Aldrich). On day 1, animals received only an i.p. injection of BPX in phosphate buffered saline solution [15]. Similarly, control (CTR) animals (*n* = 6) were immunized both on day 0 and day 1 using emulsion and pertussis toxin, but PLP_139–151_ was replaced by PBS solution.

### Animal care, clinical evaluation, and disease onset

The body weight and the clinical status of mice were monitored daily. Neurological evaluation was performed using a clinical score (CS), assigned according to the following scale: 0, no abnormality; 0.5, limp tail tip; 1, limp tail; 1.5, hind leg inhibition; 2, weakness of hind legs; 2.5, dragging of hind legs; 3, paralysis of hind legs; 3.5, hind legs are completely paralyzed and when placed on the side the mouse is unable to right itself; and 4, complete hind leg and partial front leg paralysis. Animals with initial symptoms were isolated in separate cages with easier access to food and water, and dehydrated animals received an intraperitoneal injection of saline solution at least once a day. Mice were euthanized after reaching a score of 4.

### Radiochemical synthesis

All reagents and solvents were purchased from Sigma-Aldrich Corporation. No-carrier-added ^18^F (half-life 109.8 min) was produced via the [^18^O(p,n)^18^F] nuclear reaction by a General Electric MINItrace cyclotron (10 MeV proton beam). ^18^F-fluorine at the end of a 25-μAh, 60-min (12.5 μAh) irradiation was 16–17 GBq (732–769 mCi). [^18^F]DPA-714 was labelled with ^18^F-fluoride starting from the corresponding precursor using a tosyloxy-for-fluorine nucleophilic aliphatic substitution as previously described with slight modification [16]. The specific activity at the end of synthesis and the radiochemical purity of [^18^F]DPA-714 obtained within 90 min of radiosynthesis were 300 GBq/μmol and *N* > 99%, respectively.

### PET-CT acquisition

Symptomatic EAE mice (*n* = 7) and CTR mice (*n* = 5) underwent PET/CT studies using high-resolution scanner (GE Healthcare eXplore Vista; PET resolution 1.8 mm FWHM, sensitivity 4.2% ACS, energy windows 250–700 keV; CT 200 μA, 35 kVp, resolution 200 μm) under inhalational anaesthesia (isoflurane 2%, oxygen 2 l/min). Images were acquired in dynamic mode (frame sequence 6 × 5 min) over 30 min starting 20 min after injection via tail vein of 5.55–7.00 MBq (specific radioactivity 200–800 GBq/μmol) of [^18^F]DPA-714. Images were processed using a 2D FORE/3D OSEM iterative algorithm (voxel size 0.3875 × 0.3875 × 0.775 mm) including random, scatter, dead time, and decay correction. Count rates were converted to standardized uptake values (SUV) tissue activity (MBq/cm^3^)/(injected dose (MBq)/body weight (g)). PET frames acquired between 20 and 50 min were summed and used for data analysis.

### MRI acquisition

The same animals that performed PET/CT studies underwent SPIO-MRI studies 24 h later. The MRI contrast agent FeraSpin™ R-Type (SPIO nanoparticles; iron content 500-mM Fe; mean particle size 60 nm; particle size range 10–90 nm—nanoPET Pharma GmbH, Germany) was administered via tail vein (100 μl) immediately after the injection of [^18^F]DPA-714. MRI brain acquisition was performed on a 9.4T scanner (Bruker BioSpec 94/20 USR; diameter of clear bore 200 mm, gradient amplitude 440 mT/m, maximum slew rate 3440 T/m/s), under intraperitoneal anaesthesia (Avertin 200 mg/kg). Spinal cord was not analyzed to avoid artefacts due to thoracic breathing movement.

MRI imaging protocol included a spoiled 3D gradient-echo (GRE) sequence (repetition time (TR) = 45 ms; echo time (TE) = 2.6 ms; flip angle (FA) = 15°; voxel size = 0.12 × 0.12 × 0.16 mm^3^; acquisition matrix size = [160, 96, 128]; pixel bandwidth = 248 Hz/pix) to obtain T2*-weighted images, and a 2D fast spin echo (RARE) sequence (TR = 6127 ms; TE = 37.5 ms; echo train length = 8; voxel size = 0.08 × 0.08 × 0.35 mm^3^; acquisition matrix size = [240, 216]; number of slices = 50; pixel bandwidth = 134 Hz/pix) to obtain T2-weighted images.

### PET and MRI image analysis

For each mouse, co-registration of the MRI to PET/CT was preliminarily achieved in 3dSlicer (https://www.slicer.org) [17], using the CT as proxy for PET, manually identifying six fiducial landmarks (eyes, mandibular condyles, and cochleae) on both the CT and MRI volumes. The rigid body co-registration matrix minimizing the root mean square distance was then calculated and applied to the MRI volume. Volume of interest (VOI)-based analysis of TSPO-PET and co-registered SPIO-MRI studies were then carried out based on a publicly available adult mouse brain atlas [18], which is provided in register with a CT volume. To this aim, each mouse CT was spatially normalized to the atlas CT using SPM (https://www.fil.ion.ucl.ac.uk/spm/), and the resulting normalization matrix was applied to the PET and the co-registered MRI volumes.

For each VOI, mean SUV was calculated from the PET images, while to derive estimates of the corresponding SPIO uptake, the processing pipeline was expanded, adapting the SPM tool for correction of signal inhomogeneities of SPIO images due to a non-ideal receiver coil profile and developing two dedicated software modules, respectively, for automated brain extraction and automated labelling of SPIO uptake sites by image thresholding based on the signal intensity histogram pattern (written in MATLAB® Release 2016b, The Math Works, Inc., Natick, MA, USA), thus providing fractional volumes of SPIO uptake images (SPIO-Vol).

From the VOI set available in the atlas, six regions potentially involved in the PLP-EAE model [13] were processed for the present data analysis: the cerebellum, the brainstem, the hippocampus, the caudate/putamen, the thalamus, and the neocortex. In addition, the brainstem VOI including the midbrain, pons, and medulla, not present in the atlas, was manually defined using the PMOD 3.8 v (PMOD Technologies Ltd., Switzerland) software on the coronal T2-weighted normalized MRI images. PET VOI data were normalized to the neocortex, or unaffected neocortex in mice with evidence of cortical lesions (SUVR).

### Immunofluorescence and confocal microscopy

Ex vivo immunofluorescence analysis was carried out in 5 of the EAE mice (CS from 1.5 to 3.5) and in 4 CTR mice. (3 CTR had performed PET and MRI exams, while one CTR was used only for immunofluorescence).

Mice were sacrificed at the end of the MRI study. Animals were deeply anaesthetized, and all detailed information about transcardial perfusion, brain and spinal cord isolation, tissue processing, incubation with specific antibodies, and image acquisition are previously described [19].

The following CNS regions were studied: prefrontal cortex, motor cortex, striatum, thalamus, hippocampus, corpus callosum, brainstem (midbrain, pons, and medulla), cerebellum, and spinal cord (cervical and thoracolumbar regions). For a correct identification of all regions, the mouse brain atlas of Paxinos and Franklin was used for guidance [20].

In addition, quantitative analysis of TSPO and Iba1 fluorescence intensities on tissue sections was performed at the level of cerebellum, medulla, and spinal cord using the ImageJ software (NIH, Bethesda, MA, USA), as described previously [21]. Images were acquired in brain parenchima and in proximity to the perivascular space, but distant from the perivascular bulk. Each individual value of fluorescence intensities in the EAE group was normalized to the averaged value obtained in the same region in the control group and expressed as percentage.

The quantification of co-localization between TSPO and Iba1 immunostaining was assessed by using the ‘co-localization highlighter’ plug-in for the ImageJ software (NIH, Bethesda, MA, USA). Before co-localization analysis, images were first thresholded to identify the positive signal; subsequently, the pixels expressing both TSPO and Iba1 were identified. Finally, the number of pixels positive for both TSPO and Iba1 was measured per microscope field, as previously described [22]. This value, expressed as percentage of co-localization, represents the extent of TSPO expression in Iba1-positive macrophage/microglia. Four sections from each area were analyzed, with *n* = 4 mice per group (EAE and CTR), for a total of 16 sections per group. For quantification, 4 EAE mice with similar CS were used (range 2.5–3.5).

### Iron staining and immunohistochemistry

Histologic examination of SPIO particles was performed on brain and spinal cord sections from 5 symptomatic EAE and 2 CTR mice which underwent PET and MRI using Prussian blue iron staining with the Accustain Iron Kit (Sigma-Aldrich). Sections were stained with a solution containing equal parts of 4% potassium ferrocyanide and 1.2-mmol/ HCl. Nuclei were counterstained in red/pink colour with pararosaniline solution.

To visualize co-expression of SPIO within microglia/macrophages or TSPO, in one CTR and in one EAE mouse, sections at the level of the brainstem, cerebellum, and spinal cord were first incubated with the mouse monoclonal antibody anti-F4/80 conjugated to biotin (1:400; Bio-Rad) or rabbit polyclonal antibody anti-TSPO (1:500) [23]. F4/80 antibody was used since it is more suitable than anti-Iba1 antibody for immunohistochemical analysis in non-paraffined sections in combination with Prussian blue staining as previously reported [24]. Then, immunosignals were detected with the HRP-DAB (3,3-diaminobenzidine) staining kit (UltraTek HRP; ScyTek Laboratories). Finally, the slices were counterstained with Prussian blue for the identification of iron deposits. Images were acquired with a Zeiss LSM700 microscope using × 10 and × 63 magnification. The number of TSPO^+^, SPIO^+^, F4/80^+^, TSPO/SPIO^+^, and F4/80/SPIO^+^ cells was determined in the perivascular region by manual counting at × 60 magnification and at a distance of 60 μm from the blood vessel border. Only cells with a clearly visible cell body were included.

### Statistical analysis

The non-parametric Mann-Whitney test was used for statistical comparison of changes in weight, SPIO-Vol, SUVR, and immunohistochemistry fluorescence between symptomatic EAE and CTR groups. Spearman’s rank analysis was used to determine the degree of correlation between USPIO-Vol, PET-SUVR, and clinical scores including both EAE and control mice. The level of significance was set at *p* < 0.05. The statistical analysis was performed with PASW 18 (SPSS statistics).

## Results

### Clinical characteristics

All immunized mice developed clinical signs of EAE. The first clinical signs occurred between 9 and 14 days after PLP_139–151_ immunization with a CS ranging from 0.5 to 2 and then quickly raised reaching peak values of 1.5 to 3.5 between 12 and 15 days. Compared with CTR, the averaged body weight in EAE mice started to significantly decrease from day 11 (mean ± SD 19.63 ± 0.83 g vs 17.14 ± 1.69 g, *p* < 0.05). PET studies were performed in symptomatic mice between 11 and 14 days post-immunization (mean ± SD 12.43 ± 1.4). At PET study, in EAE mice, CS ranged from 1 to 2 (mean ± SD 1.64 ± 0.38). At MRI study, the day after the PET exam, the CS ranged from 1 to 3.5 (mean ± SD 2.36 ± 0.93). Control mice underwent PET between 11 and 13 days post-immunization (mean ± SD 11.5 ± 1).

### [^18^F]DPA-714-PET

**Fig. 1:**
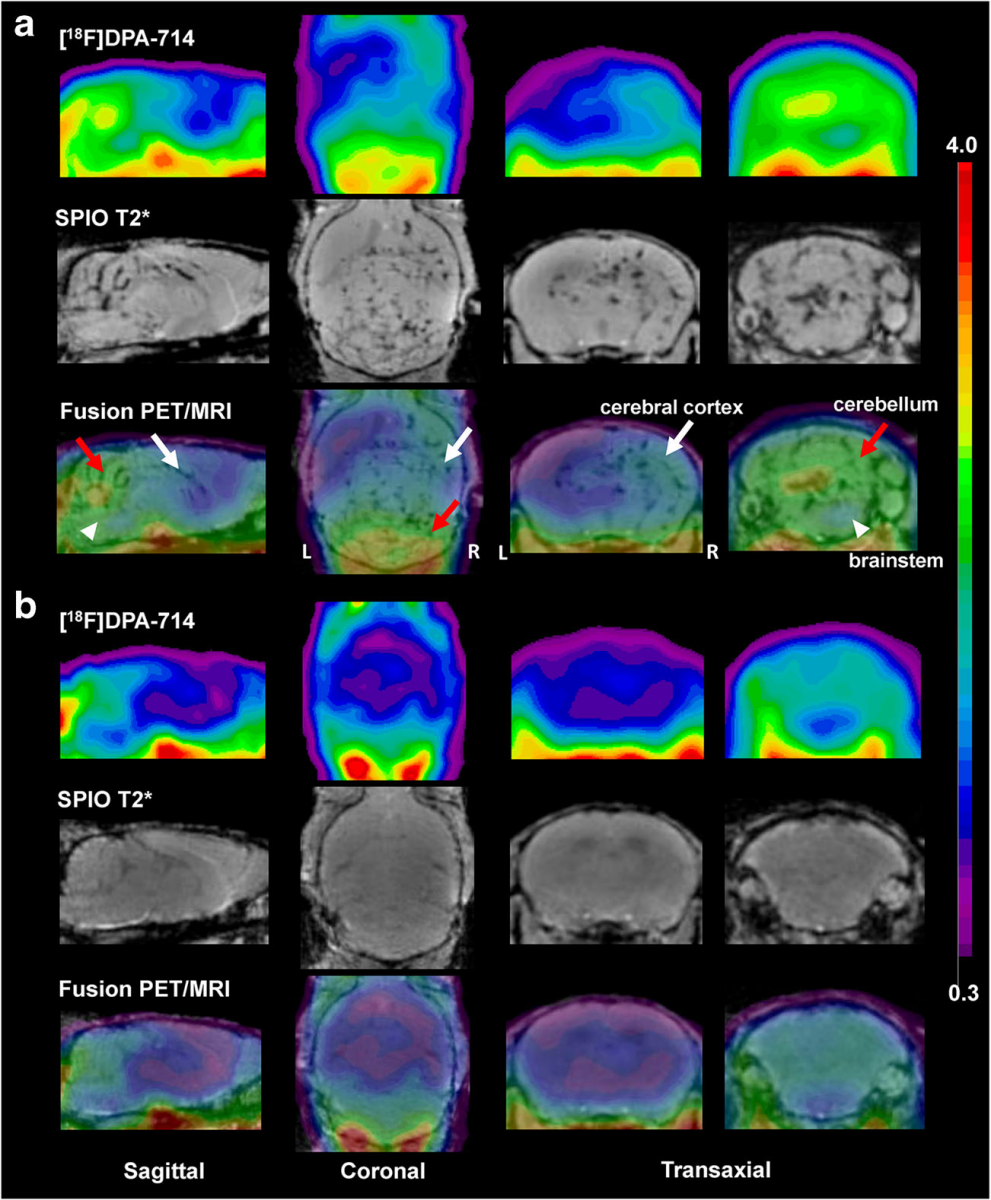
Sagittal, coronal, and transaxial [^18^F]DPA-714 PET, SPIO T2*MRI, and PET/MRI fusion images of a representative EAE mouse (CS 2.5) (**a**) and control (**b**). PET images represent summed scans (20–50 min post-injection) normalized to the left cerebral neocortex SUV values. Increased radiotracer uptake and loss of T2* signal can be observed in the cerebellum (red arrow), brainstem (white arrowheads), and, to a lesser extent, right cerebral cortex (white arrow) in the EAE mouse but not in the control. R, right; L, left

**Fig. 2:**
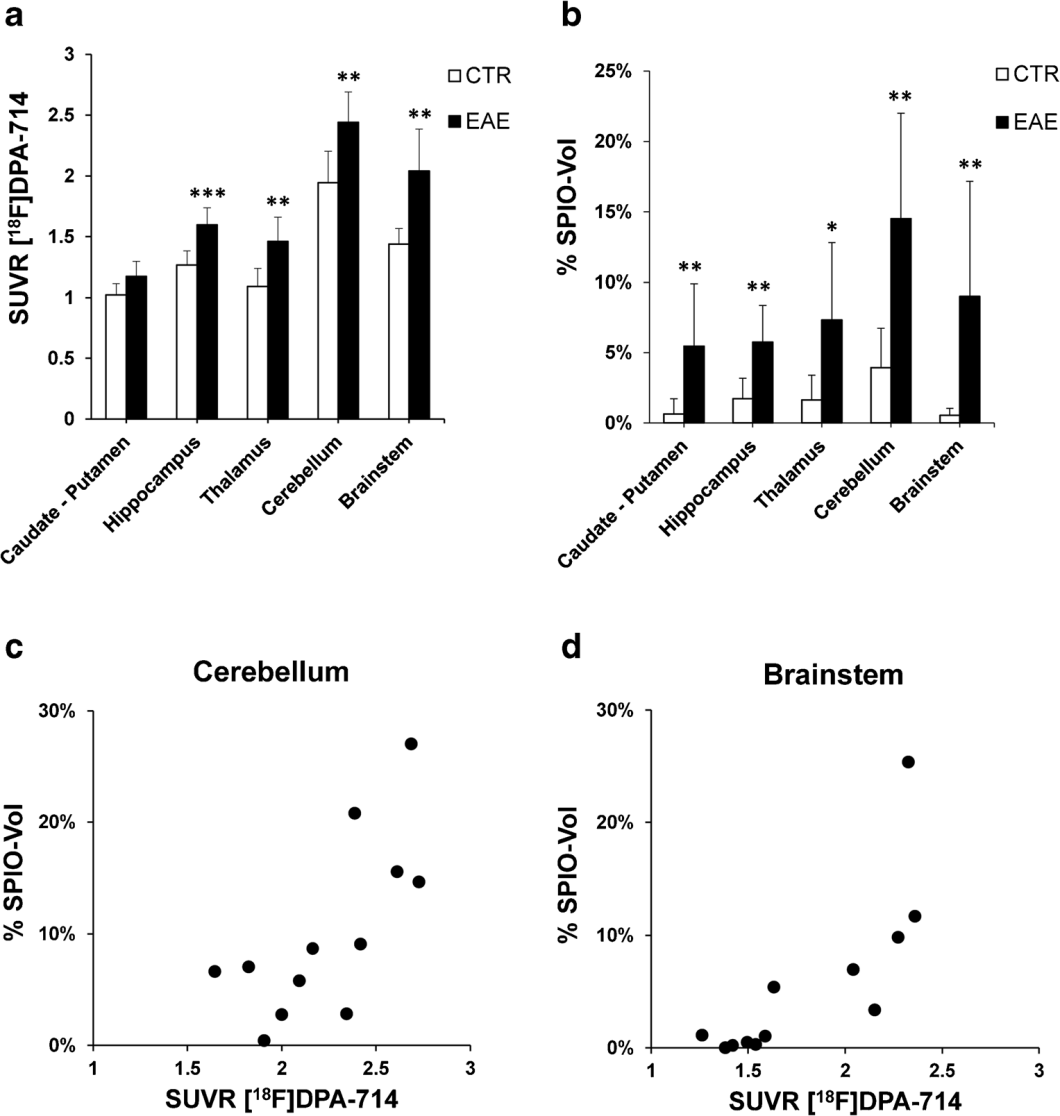
[^18^F]DPA-714 SUVR (**a**) and SPIO-Vol values (**b**) (mean ± SD) show a significant increase in the different brain regions in EAE mice compared with controls (mean ± SD); *p < 0.05, **p < 0.02, ***p < 0.01. Scatterplots show a positive significant correlation between [^18^F]DPA-714 SUVR and SPIO-Vol in the cerebellum (rho 0.727, *p* < 0.01) (**c**) and the brainstem (rho 0.860, *p* < 0.001) (**d**)

Visual assessment of PET images revealed higher uptake of [^18^F]DPA-714 in the brainstem and cerebellum in symptomatic EAE mice as compared with CTR in most part of animals, spreading to subcortical and, to a lesser extent, to neocortical regions of the right brain hemisphere in 2 EAE mice (Fig. 1a, b). Averaged SUV in the neocortex was not significantly different in EAE as compared with CTR (0.207 ± 0.090 and 0.225 ± 0.105, respectively; *p* = 0.787). Regional SUVR (mean ± SD) measured in EAE and CTR is summarized in Fig. 2a and Table 1. The mean SUVR values were significantly increased in EAE mice compared with that in CTR in the hippocampus (*p* < 0.01), thalamus, cerebellum, and brainstem (*p* < 0.02)*.* Significant positive correlation was found between CS and SUVR in the hippocampus and the brainstem (rho 0.839, *p* < 0.002), the cerebellum (rho 0.810, *p* < 0.002), the thalamus (rho 0.861, *p* < 0.001), and the caudate/putamen (rho 0.714, *p* < 0.01).

**Table 1:** SUVR and SPIO-Vol (mean ± SD) in different CNS regions in EAE and CTR

Region	SUVR [^18^F]DPA-714		% SPIO-Vol	
	EAE	CTR	EAE	CTR
Caudate–Putamen	1.2 ± 0.1	1.0 ± 0.1	5.4 ± 4.5**	0.6 ± 1.1
Hippocampus	1.6 ± 0.1***	1.3 ± 0.1	5.7 ± 2.6**	1.7 ± 1.5
Thalamus	1.5 ± 0.2**	1.1 ± 0.1	7.3 ± 5.5*	1.6 ± 1.7
Cerebellum	2.4 ± 0.2**	1.9 ± 0.3	14.5 ± 7.5**	3.9 ± 2.8
Brainstem	2.0 ± 0.3**	1.4 ± 0.1	9.0 ± 8.1**	0.5 ± 0.5

*p < 0.05

**p < 0.02

***p < 0.01

### SPIO-MRI

In EAE mice, punctate susceptibility signals were observed at T2*-weighted images in the cerebellum in all animals, and in the brainstem, the cerebral cortex, the striatum, and the thalamus in 5/7 animals. Overall, 2/7 mice showed the most widespread and marked increase in SPIO susceptibility signal involving the cerebellum, the brainstem, the striatum, the thalamus bilaterally, and the cerebral cortex of one side (Fig. 1a). In CTR, there was no evidence of increased signal (Fig. 1b). Averaged SPIO-Vol values were significantly increased in the hippocampus, cerebellum, caudate/putamen, brainstem (*p* < 0.02), and, to a lesser extent, thalamus (*p* < 0.05) (Fig. 2b, Table 1). No significant difference between EAE and CTR was found in the neocortex. SPIO-Vol values were positively correlated with CS in the brainstem, the cerebellum, the thalamus, and the hippocampus (*p* < 0.01).

### [^18^F]DPA-714-PET and SPIO-MRI

At visual inspection, the topography and the extent of increased [^18^F]DPA-714 uptake and susceptibility signal at T2* MRI were similar in almost all animals. This was also particularly evident in the two mice with widespread and asymmetric involvement of different brain regions (Fig. 1a). These visual findings are supported by quantitative data showing significant positive correlation between [^18^F]DPA-714 SUVR and SPIO-Vol in the hippocampus (rho 0.727, *p* < 0.01), thalamus (rho 0.671, *p* < 0.05), cerebellum (rho 0.727, *p* < 0.01), and brainstem (rho 0.860, *p* < 0.001) (Fig. 2c, d).

### Immunohistochemistry

**Fig. 3:**
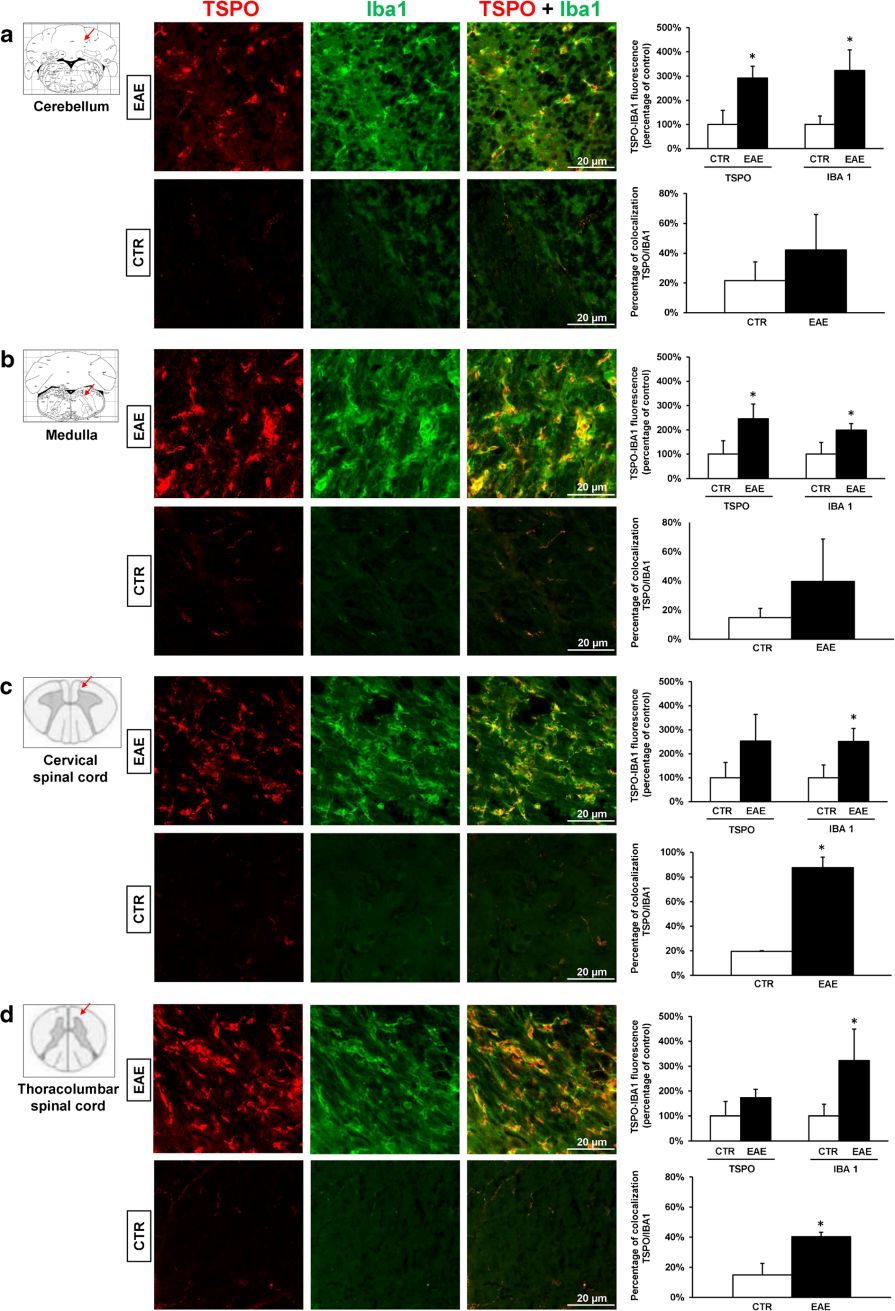
Confocal images of double-labelled immunofluorescence for TSPO (red), Iba1 (green), and merged TSPO + Iba1 (orange) in the cerebellum (**a**), medulla (**b**), cervical (**c**), and thoracolumbar spinal cord (**d**) in the EAE and control reported in Fig. 1. The histograms represent quantitative TSPO and Iba1 fluorescence intensity and TSPO/Iba1 co-localization (mean ± SD) in the different regions. *p ≤ 0.05 for EAE vs control. Scale bar 20 μm

Confocal microscopy analysis revealed high increase of both TSPO and Iba1 fluorescence intensities in the cerebellum, medulla, and spinal cord of all EAE mice as compared with CTR mice. In the two mice with higher CS (2.5 and 3.5) and showing at both PET and MRI, the most widespread and marked increase in TSPO and SPIO brain uptake and increased Iba1 and TSPO immunosignals were also evident in the motor cortex, hippocampus, striatum, and thalamus. The increased immunoreactivity appeared less marked in EAE with lower (1.5) than with higher clinical score in all regions. Double-immunofluorescence analysis detected a clear co-localization between TSPO and Iba1-positive cells mainly with a round shape (Fig. 3).

Quantitative confocal double-immunofluorescence analyses showed increased TSPO and Iba1 fluorescence intensities in EAE mice compared with CTR in all regions examined. Statistical analysis (Table 2, Fig. 3) revealed significant increased expression of TSPO and Iba1 in the cerebellum (Fig. 3a) and medulla (Fig. 3b), and only of Iba1 (*p* < 0.05) in cervical (Fig. 3c) and thoracolumbar (Fig. 3d) spinal cord tracts in EAE compared with CTR. Significant increase in co-expression of TSPO with Iba1-positive cells was found in the cervical (Fig. 3c) and thoracolumbar (Fig. 3d) spinal tracts of EAE compared with CTR. In all these regions, double-labelled TSPO-Iba1-positive cells showed enlarged cell bodies and shortened cellular processes (Fig. 3).

**Table 2:** TSPO, Iba1 fluorescence, and TSPO/Iba1 co-localization (mean ± SD) in different CNS regions in EAE and CTR

Region	TSPO fluorescence intensity (%)		Iba1 fluorescence intensity (%)		TSPO/Iba1 co-localization (%)	
	EAE	CTR	EAE	CTR	EAE	CTR
Cerebellum	292 ± 49*	100 ± 59	324 ± 85*	100 ± 35	42 ± 24	22 ± 13
Medulla	245 ± 61*	100 ± 55	199 ± 27*	100 ± 47	40 ± 29	15 ± 6
Cervical spinal cord	253 ± 111	100 ± 63	252 ± 54*	100 ± 53	88 ± 15*	20 ± 0.5
Thoracolumbar spinal cord	173 ± 34	100 ± 58	322 ± 127*	100 ± 47	40 ± 5*	15 ± 13

*p < 0.05

Iron deposits were observed in perivascular regions of the cerebellum and medulla in all EAE mice examined and in the spinal cord of the 2 mice with the highest CS (3.5 and 2.5). In these latter, a minimal presence of iron deposits was observed in the corpus callosum, striatum, hippocampus, and thalamus, and only in one (CS 2.5) in motor and prefrontal cortices. Few blue spots were also present in the corpus callosum of a symptomatic mouse with CS of 1.5. No iron deposits were observed in CTR mice.

**Fig. 4:**
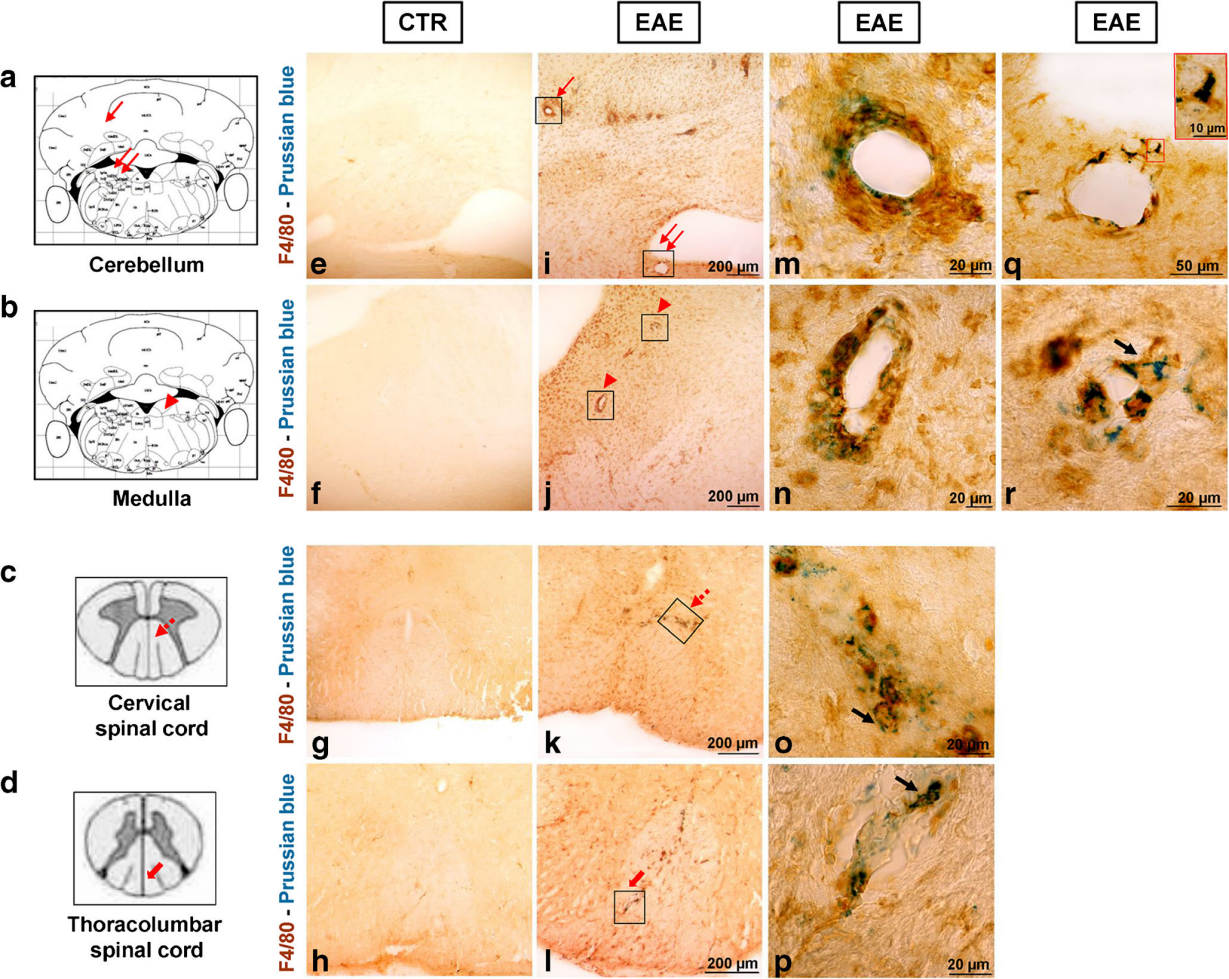
F4/80 (brown) and Prussian blue staining in brain sections at the level of the cerebellum (**a**), medulla (**b**), cervical (**c**), and thoracolumbar spinal cord (**d**) from control (**e**–**h**) and EAE mouse (**i**–**r**) reported in Fig. 1. Higher-magnification images of the frame depicted in **i**–**l** displaying double-labelled microglia/macrophages in perivascular regions at level of primary fissure (**m**), area indicated with red arrow in **i**; spinal vestibular nucleus (**q**), region indicated with double red arrows in **i**; parvicellular part of medial vestibular nucleus (**n** and **r**), area pointed with arrowhead in **j**; white matter of spinal cord (**o** and **p**) in correspondence of dashed arrow and large arrow in **k** and **l** respectively. Higher magnification of the frame depicted in **q** shows a single double-labelled cells. Arrows in panels **r**, **o**, and **p** point to double-labelled cells. Arrows indicating the different regions are also reported in the left schematic panels (**a**–**d**). Scale bars in **e**–**l** 200 μm, in **m**–**r** 20 μm, in **q** 50 μm

**Fig. 5:**
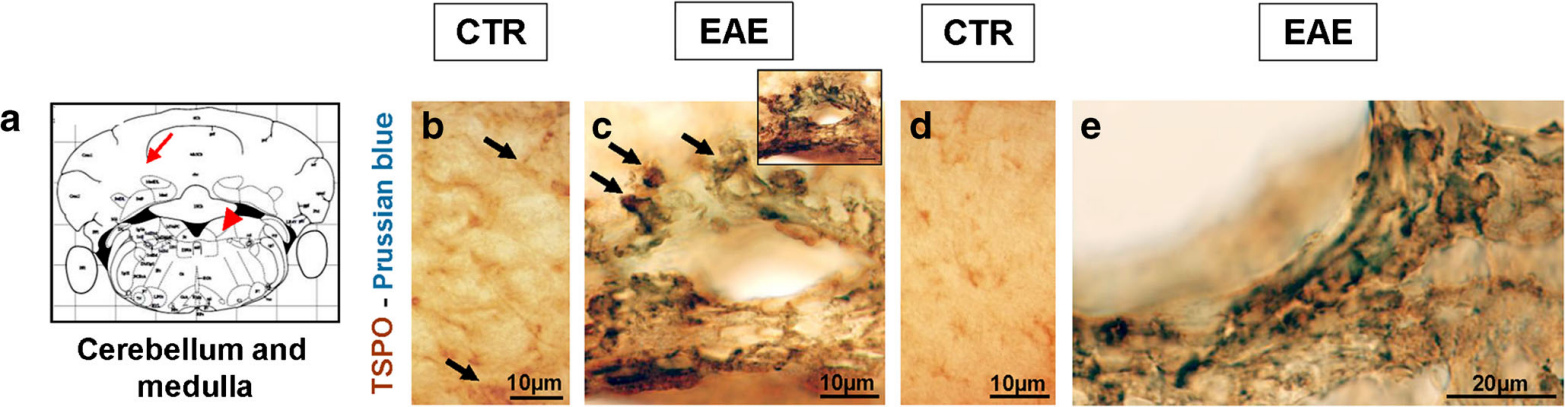
Distribution of TSPO (brown) and Prussian blue staining in the cerebellum (**a**) (red arrow) and medulla (**a**) (arrowhead). Light microscopy images displaying TSPO, iron-positive cells, and TSPO/iron double-labelled cells in brain sections from control (**b**, **d**) and EAE mice (**c**, **e**). Arrows point to perivascular double-labelled TSPO/iron cells at the level of primary fissure in cerebellum (**c**) and of parvicellular part of medial vestibular nucleus in medulla (**e**) of the symptomatic EAE. Scale bars in **b**–**d** 10 μm, in **e** 20 μm

Combined F4/80 antibody and Prussian blue staining revealed a very pronounced immunosignal of both F4/80 and iron staining confined in areas of dense hypercellularity (plaques) scattered in the cerebellum and medulla and within white matter regions of both cervical and thoracolumbar spinal cord (Fig. 4). Interestingly, higher-magnification images revealed co-localization of iron particles within activated microglia/macrophage cells in the perivascular space (Fig. 4m, n, q, r) or in the white matter spinal cord (Fig. 4o, p). A similar distribution was observed for iron and TSPO. Indeed, expression of TSPO in SPIO-loaded cells infiltrating the brain parenchyma of EAE-PLP mice was observed in areas of dense perivascular hypercellularity within cerebellum and medulla (Fig. 5). Quantitative co-expression analyses of TSPO/iron-positive or F4/80/iron-positive cells revealed that 68 ± 8.9% and 66 ± 7.3% of perivascular cells showing iron staining also co-expressed TSPO protein or F4/80 marker, respectively.

## Discussion

In this study, by combining PET with [^18^F]DPA-714 and MRI with SPIO, we were able to track non-invasively the spatial distribution of brain microglial/macrophage activation in a mouse model of RR-MS. Significant increased average uptake of [^18^F]DPA-714 and SPIO was observed in the brainstem, cerebellum, hippocampus, and thalamus in PLP-EAE symptomatic mice, compared with controls, during the acute phase. Immunostaining revealed increased TSPO expression and iron deposits in those regions displaying higher uptake and co-localization with the microglia/macrophage markers Iba1 and F4/80. These findings suggest that both resident microglia and infiltrating blood-derived macrophages contribute to neuroinflammation processes in the same vulnerable brain regions at this stage of EAE.

In contrast to other models of EAE, mainly affecting the spinal cord, the PLP-immunized SJL/J mice have the advantage of exhibiting also widespread brain pathology [12] similar to that reported in human MS and measurable by in vivo brain imaging. Typical lesions are reported in neuropathological studies in the brainstem, the cerebellum [25], and the forebrain white and grey matter [26]. Profound activation of resident microglia and blood-derived macrophage infiltration characterize the neuroinflammatory lesions [12, 26] and might be differently involved in the pathogenesis of EAE [5].

Despite this interest, only a few TSPO-PET and (U)SPIO-MRI studies have investigated the brain neuroinflammatory changes in PLP-EAE mice, and to our knowledge, this is the first study in which both methods were combined to assess their sensitivity and possible complementarities or differences in tracking microglia/macrophage brain regional changes in this EAE model with widespread brain pathology.

Our PET results are in part in line with the strong increase of [^18^F]PBR111 uptake reported ex vivo and in vivo with PET in several brain regions during the acute phase of PLP-induced EAE in SJL mice [13]. In particular, the ex vivo data [13] showed that the cerebellum and the hippocampus were affected very early even before the onset of symptoms, suggesting high susceptibility to neuroinflammation of this region, a hypothesis supported in the cerebellum by neuropathological data demonstrating early breakdown of cerebellar BBB prior of the thoracolumbar spinal tract [27]. Interestingly, the vulnerability of the cerebellum and the brainstem has been shown also in other EAE murine models studied with different TSPO radiotracers. The [^11^C]PK11195 binding was increased in the cerebellum of myelin basic protein (MBP)-EAE rats [28] and in the brainstem of myelin oligodendrocyte glycoprotein (MOG)-EAE rats [29], while the [^18^F]VC701 binding was increased in the cerebellum and the midbrain of MOG-EAE mice [30]. Differently from Mattner and co-authors [13], we did not find a significant increase of [^18^F]DPA-714 binding in the cortex and in the striatum in the group of EAE mice compared with controls. However, widespread cortical and subcortical radiotracer uptake was observed in 2 animals with more severe disease, suggesting possible heterogeneity among animals.

Our immunohistochemistry and confocal double-immunofluorescence results showed that during the peak stage of the first RR-EAE episode in PLP-immunized mice, the expression of both TSPO and the microglia/macrophage marker Iba1 as well as their co-expression increased in different brain regions including cerebellum, brainstem, and spinal cord. Indeed, during this stage, increased activated microglia/macrophages are related to increased TSPO expression, in line with previous report in the same model [13].

Interestingly, our quantitative analyses revealed that both TSPO and Iba1 fluorescence were particularly increased in the cerebellum and in the midbrain. Co-expression studies indicate however that they co-localized with higher extent in the cervical and thoracolumbar spinal tracts, and to a lesser extent in the cerebellum and in the midbrain, thus suggesting, in line with previous studies, that TSPO may be not exclusively expressed by microglia/macrophages in inflamed tissue [4, 6, 7, 31]. On the other hand, it should be considered that our co-localization analyses, expressed as percentage of overlapping pixels between TSPO and Iba1 immunostaining, represent the extent (or magnitude) of TSPO expression in Iba1-positive microglia. This quantitative approach was preferred over cell counting for the following reasons: 1) cell counting is subject to user bias, as the investigator makes a choice about whether a cell profile should be counted; 2) cell counting of microglia/macrophage profiles is often very difficult in highly inflamed EAE tissue, because resident and infiltrating inflammatory cells form a densely packed layers that often overlap or fuse together; 3) the function of microglia is not always predicted by cell number but by how much a selected antigen is up- or downregulated. In this regard, the upregulation of Iba1 is largely recognized as hallmark of microglia activation in different neuropathological conditions [32]. Based on these considerations, we cannot exclude the possibility that as the subcellular target of antibodies differ (mitochondria for TSPO, more diffuse for Iba1), our analysis may lead to an underestimation of co-localization, missing pixel with no co-localization in cells expressing both markers.

Indeed, TSPO expression has been shown to be significantly increased in reactive astrocytes during neuroinflammation in different neuropathological conditions [4]. Although the increased TSPO expression seems not to be associated with GFAP-positive cells at the first clinical episode in PLP-EAE mice [13], Daugherty et al. [33], by employing transgenic mice in which TSPO was deleted in astrocytes, oligodendrocytes, and ependymal cells, clearly showed that TSPO in these cells may play a role in neuroinflammation during EAE. Further studies will be required to better characterize TSPO distribution in different brain cell populations and cerebral regions, and at different time points during EAE.

One of the strengths of our study was to measure SPIO accumulation and [^18^F]DPA-714 uptake in the same animal and in the same brain regions. MR images obtained in the same mice revealed a regional pattern of brain SPIO accumulation similar to that observed in [^18^F]DPA-714-PET images as supported by visual and semi-quantitative analysis of co-registered PET-MRI images. Prominent and significant increase in SPIO load was found in the cerebellum and the brainstem, but also in the hippocampus, the caudate/putamen, and, to a lesser extent, the thalamus in EAE mice as compared with controls. Moreover, [^18^F]DPA-714 uptake and SPIO accumulation were positively correlated in the same regions except in the striatum where a prominent SPIO accumulation seems to occur. As for PET results, SPIO load was less evident in the cerebral cortex except in the two mice also showing widespread cortical-subcortical [^18^F]DPA-714 uptake. The MR findings are in agreement with a previous in vivo and ex vivo study in the same EAE mouse model that has shown increased susceptibility signal in the cerebellum GM and WM, midbrain, basal ganglia, and, to a lesser extent, cerebral cortex in the acute phase of disease 48 h after administration of cross-linked iron oxide nanoparticles (CLIOs) [12]. Interestingly, the cerebellum and the brainstem were the regions also showing most frequently increased SPIO accumulation in our EAE symptomatic mice even at an individual level in line with data reported in the same model [12] and different rodent models of EAE using different MR field strengths [34–37].Our immunohistochemical data showed iron particles within F4/80- and TSPO-positive cells mainly located in perivascular space in the cerebellum and medulla. We also studied by immunohistochemistry the TSPO expression and the iron accumulation in the spinal cord, a main target of the PLP-EAE mouse model. We did not apply in vivo imaging because the low spatial resolution of small animal PET would have hampered the accurate in vivo measurement of TSPO sites in this region. Both spinal tracts also showed areas of hypercellularity positive for Prussian blue and TSPO or F4/80 immunostaining. Relevantly, in all brain regions examined, Prussian blue-positive cells were confined predominantly, if not only, along the perivascular space. These findings suggest that SPIO could associate mainly with perivascular inflammation, whereas TSPO could traduce both perivascular and parenchymal inflammation.

Finally, we demonstrated that worsening of clinical score was significantly correlated with increased [^18^F]DPA-714 binding and SPIO accumulation in the brainstem, the cerebellum, the hippocampus, and the thalamus and with increased [^18^F]DPA-714 binding in the striatum underlying the role of these neuroinflammatory changes in disease severity.

The interpretation of our results with respect to the cellular contribution to [^18^F]DPA-714-PET and SPIO-MRI signals is complex. It is still debated and experimentally difficult to demonstrate whether SPIOs are taken up by resident macrophages derived from activated microglia or by haematogenous monocyte-derived macrophages that infiltrate the CNS, since both share many surface markers. Previous studies in transgenic MOG-EAE mice suggest that the iron particles are specifically localized in EGFP (enhanced green fluorescent protein)-positive haematogenous macrophages but not in EGFP-negative microglia in inflammatory brain lesions [38]. On the other hand, flow cytometry analysis performed in the cerebellum and in the cerebrum of the PLP-EAE mice showed that infiltrating macrophages and resident microglia took up CLIO particles [12].

Previous studies employing in vivo experimental models of MS demonstrated that TSPO proteins are expressed by both CNS resident and circulating innate immune cells [31]. Hence, also in our model, it is plausible that our confocal co-localization studies with TSPO and Iba1 antibodies may reflect TSPO expression not only in microglia but also in infiltrating blood-derived macrophages. In support of this hypothesis, we found that SPIO-positive cells were mostly detected along perivascular space, where infiltrating cells were clearly identifiable. Interestingly, we found that a significant proportion of them (about 70%) also co-expressed TSPO or the microglia/macrophage marker F4/80. Altogether, these findings, beside suggesting the presence of the translocator protein in infiltrating blood-derived SPIO-loaded macrophages, also may indicate that a significant proportion of TSPO-positive cells in the perivascular space may belong to monocyte-derived microglia/macrophages. Future studies using more specific markers recently developed that allow to differentiate resident microglia/microphages from infiltrating blood-derived macrophages, such as TMEM119 [39] or distinguish the phenotype of M1/M2 microglia/macrophage subsets, will provide new insights on the contribution of each cell type to the in vivo signal of SPIO-MRI and TSPO-PET.

Overall, our results suggest that in this model and at this stage of disease, both resident-activated microglia/macrophage and macrophage infiltrations might contribute to the inflammatory processes in specific susceptible brain regions and might have a detrimental role as suggested by the strong correlation with disease severity. A prevalent vulnerability to neuroinflammation of the cerebellum and brainstem is suggested, while the involvement of other regions might be more heterogeneous among animals.

PET and MRI imaging of microglia/macrophage in the brain are emerging non-invasive techniques that have complementary sensitivity and spatial resolution. PET exceeds MRI for sensitivity, allowing the measure of small concentrations of the molecular target in the brain, while MRI shows excellent spatial resolution as compared with PET, providing precise information on the cell trafficking in small brain region such as the brainstem and cortical grey matter/white matter regions, of particular relevance in mice studies.

Current results do not show differences in the patterns of these two imaging approaches, at least in the acute stage of the first relapse in PLP-EAE. However, given the potential differences in the targeted cell populations (mainly limited to peripheral monocytes and microglia for SPIO, while DPA-714 uptake is likely to be influenced also by other CNS cell populations), longitudinal studies, covering different clinical stages, are warranted before drawing firm conclusions on the relative merits of the two techniques.

The main limitation of this study was the small number of mice. Thus, caution is required in the interpretation of these results although our TSPO-PET and SPIO-MRI findings are in agreement with those reported in the literature in separate studies.

Moreover, in this study, we have not evaluated the status of BBB. This is a critical point when BBB alterations are present and might result in passive entry of the radiotracer and SPIO into the brain. Previous ex vivo and in vivo competition studies in the same mouse model, however, demonstrated the specificity of [^18^F]PBR111 binding, another second-generation TSPO radiotracer similar to [^18^F]DPA-714, in the same regions [13]. Moreover, previous results suggest that increased BBB permeability, as imaged by Gd DTPA-enhanced MRI, does not always explain USPIO brain uptake because it may occur prior to USPIO load, is not correlated with the presence of cellular infiltrates in the brain [34, 37], is weakly correlated with clinical score [12], and may display a regional distribution different from that of USPIO accumulation [36].

In conclusion, despite these limitations, our study demonstrates the sensitivity of the [^18^F]DPA-714 for in vivo imaging of brain microglia/macrophage activation during the acute phase in PLP-EAE mice similar to what reported in the spinal cord of MBP-EAE rats [40] and in the brain grey and white matter of cuprizone-MS mice during demyelination periods [41]. Moreover, it suggests that the combination of TSPO-PET and SPIO-MRI may represent a feasible strategy to detect and characterize in vivo the neuroinflammatory processes occurring in vulnerable brain regions in a mouse model resembling the human MS-RR. Future studies with distinct cellular markers able to distinguish the brain resident microglia from monocyte-derived macrophages or further characterization of the cellular source of in vivo TSPO-PET and SPIO-MRI signals will be instrumental to better understand the specific contribution of each of these innovative methods to the in vivo detection and monitoring of the spatiotemporal changes of these cells and their role in initiating and promoting disease progression and degeneration in view of new MS therapeutic strategies.

## Data Availability

The dataset generated during and/or analyzed during the current study are available from the corresponding author on reasonable request.

## References

[1] Calabrese M, Magliozzi R, Ciccarelli O, Geurts JJ, Reynolds R, Martin R (2015). Exploring the origins of grey matter damage in multiple sclerosis. Nat Rev Neurosci.

[2] Hemmer B, Kerschensteiner M, Korn T (2015). Role of the innate and adaptive immune responses in the course of multiple sclerosis. Lancet Neurol.

[3] Chu F, Shi M, Zheng C, Shen D, Zhu J, Zheng X (2018). The roles of macrophages and microglia in multiple sclerosis and experimental autoimmune encephalomyelitis. J Neuroimmunol.

[4] Jacobs AH, Tavitian B, InMind Consortium (2012). Noninvasive molecular imaging of neuroinflammation. J Cereb Blood Flow Metab.

[5] Ciccarelli O, Barkhof F, Bodini B, De Stefano N, Golay X, Nicolay K (2014). Pathogenesis of multiple sclerosis: insights from molecular and metabolic imaging. Lancet Neurol.

[6] Banati RB (2002). Visualising microglial activation in vivo. Glia..

[7] Betlazar C, Harrison-Brown M, Middleton RJ, Banati R, Liu GJ (2018). Cellular sources and regional variations in the expression of the neuroinflammatory marker translocator protein (TSPO) in the normal brain. Int J Mol Sci.

[8] Ugga L, Romeo V, Tedeschi E, Brunetti A, Quarantelli M (2018). Superparamagnetic iron oxide nanocolloids in MRI studies of neuroinflammation. J Neurosci Methods.

[9] Nathoo N, Yong VW, Dunn JF (2014). Understanding disease processes in multiple sclerosis through magnetic resonance imaging studies in animal models. Neuroimage Clin.

[10] Gargiulo S, Coda AR, Panico M, Gramanzini M, Moresco RM, Chalon S (2017). Molecular imaging of neuroinflammation in preclinical rodent models using positron emission tomography. Q J Nucl Med Mol Imaging.

[11] Ransohoff RM (2012). Animal models of multiple sclerosis: the good, the bad and the bottom line. Nat Neurosci.

[12] Kirschbaum K, Sonner JK, Zeller MW, Deumelandt K, Bode J, Sharma R (2016). In vivo nanoparticle imaging of innate immune cells can serve as a marker of disease severity in a model of multiple sclerosis. Proc Natl Acad Sci U S A.

[13] Mattner F, Staykova M, Berghofer P, Wong HJ, Fordham S, Callaghan P (2013). Central nervous system expression and PET imaging of the translocator protein in relapsing-remitting experimental autoimmune encephalomyelitis. J Nucl Med.

[14] Matarese G, Sanna V, Di Giacomo A, Lord GM, Howard JK, Bloom SR (2001). Leptin potentiates experimental autoimmune encephalomyelitis in SJL female mice and confers susceptibility to males. Eur J Immunol.

[15] De Rosa V, Procaccini C, La Cava A, Chieffi P, Nicoletti GF, Fontana S (2006). Leptin neutralization interferes with pathogenic T cell autoreactivity in autoimmune encephalomyelitis. J Clin Invest.

[16] Vicidomini C, Panico M, Greco A, Gargiulo S, Coda AR, Zannetti A (2015). In vivo imaging and characterization of [(18)F]DPA-714, a potential new TSPO ligand, in mouse brain and peripheral tissues using small-animal PET. Nucl Med Biol.

[17] Fedorov A, Beichel R, Kalpathy-Cramer J, Finet J, Fillion-Robin J-C, Pujol S (2012). 3D slicer as an image computing platform for the quantitative imaging network. Magn Reson Imaging.

[18] Chuang N, Mori S, Yamamoto A, Jiang H, Ye X, Xu X (2011). An MRI-based atlas and database of the developing mouse brain. Neuroimage..

[19] Gargiulo S, Anzilotti S, Coda AR, Gramanzini M, Greco A, Panico M (2016). Imaging of brain TSPO expression in a mouse model of amyotrophic lateral sclerosis with (18)F-DPA-714 and micro-PET/CT. Eur J Nucl Med Mol Imaging.

[20] Paxinos G, Franklin KB (2001). The mouse brain in stereotaxic coordinates.

[21] Anzilotti S, Brancaccio P, Simeone G, Valsecchi V, Vinciguerra A, Secondo A (2018). Preconditioning, induced by sub-toxic dose of the neurotoxin L-BMAA, delays ALS progression in mice and prevents Na+/Ca2+ exchanger 3 downregulation. Cell Death Dis.

[22] de Rosa V, Secondo A, Pannaccione A, Ciccone R, Formisano L, Guida N (2019). D-Aspartate treatment attenuates myelin damage and stimulates myelin repair. EMBO Mol Med.

[23] Nishiyama A, Yu M, Drazba JA, Tuohy VK (1997). Normal and reactive NG2+ glial cells are distinct from resting and activated microglia. J Neurosci Res.

[24] Desestret V, Brisset JC, Moucharrafie S, Devillard E, Nataf S, Honnorat J (2009). Early-stage investigations of ultrasmall superparamagnetic iron oxide-induced signal change after permanent middle cerebral artery occlusion in mice. Stroke..

[25] Constantinescu CS, Farooqi N, O'Brien K, Gran B (2011). Experimental autoimmune encephalomyelitis (EAE) as a model for multiple sclerosis (MS). Br J Pharmacol.

[26] Rasmussen S, Wang Y, Kivisäkk P, Bronson RT, Meyer M, Imitola J (2007). Persistent activation of microglia is associated with neuronal dysfunction of callosal projecting pathways and multiple sclerosis-like lesions in relapsing—remitting experimental autoimmune encephalomyelitis. Brain..

[27] Tonra JR, Reiseter BS, Kolbeck R, Nagashima K, Robertson R, Keyt B (2001). Comparison of the timing of acute blood-brain barrier breakdown to rabbit immunoglobulin G in the cerebellum and spinal cord of mice with experimental autoimmune encephalomyelitis. J Comp Neurol.

[28] Martín A, Vázquez-Villoldo N, Gómez-Vallejo V, Padro D, Soria FN, Szczupak B (2016). In vivo imaging of system xc- as a novel approach to monitor multiple sclerosis. Eur J Nucl Med Mol Imaging.

[29] de Paula FD, Vlaming ML, Copray SC, Tielen F, Anthonijsz HJ, Sijbesma JW (2014). PET imaging of disease progression and treatment effects in the experimental autoimmune encephalomyelitis rat model. J Nucl Med.

[30] Belloli S, Zanotti L, Murtaj V, Mazzon C, Di Grigoli G, Monterisi C (2018). ^18^F-VC701-PET and MRI in the in vivo neuroinflammation assessment of a mouse model of multiple sclerosis. J Neuroinflammation.

[31] Nack A, Brendel M, Nedelcu J, Daerr M, Nyamoya S, Beyer C (2019). Expression of translocator protein and [18F]-GE180 ligand uptake in multiple sclerosis animal models. Cells.

[32] Boscia F, Gala R, Pannaccione A, Secondo A, Scorziello A, Di Renzo G (2009). NCX1 expression and functional activity increase in microglia invading the infarct core. Stroke..

[33] Daugherty DJ, Chechneva O, Mayrhofer F, Deng W (2016). The hGFAP driven conditional TSPO knockout is protective in a mouse model of multiple sclerosis. Sci Rep.

[34] Floris S, Blezer EL, Schreibelt G, Döpp E, van der Pol SM, Schadee-Eestermans IL (2004). Blood-brain barrier permeability and monocyte infiltration in experimental allergic encephalomyelitis: a quantitative MRI study. Brain..

[35] Hunger M, Budinger E, Zhong K, Angenstein F (2014). Visualization of acute focal lesions in rats with experimental autoimmune encephalomyelitis by magnetic nanoparticles, comparing different MRI sequences including phase imaging. J Magn Reson Imaging.

[36] Rausch M, Hiestand P, Baumann D, Cannet C, Rudin M (2003). MRI-based monitoring of inflammation and tissue damage in acute and chronic relapsing EAE. Magn Reson Med.

[37] Brochet B, Deloire MS, Touil T, Anne O, Caillé JM, Dousset V (2006). Early macrophage MRI of inflammatory lesions predicts lesion severity and disease development in relapsing EAE. Neuroimage..

[38] Oweida AJ, Dunn EA, Karlik SJ, Dekaban GA, Foster PJ (2007). Iron-oxide labeling of hematogenous macrophages in a model of experimental autoimmune encephalomyelitis and the contribution to signal loss in fast imaging employing steady state acquisition (FIESTA) images. J Magn Reson Imaging.

[39] Bennett ML, Bennett FC, Liddelow SA, Ajami B, Zamanian JL, Fernhoff NB (2016). New tools for studying microglia in the mouse and human CNS. Proc Natl Acad Sci U S A.

[40] Abourbeh G, Thézé B, Maroy R, Dubois A, Brulon V, Fontyn Y (2012). Imaging microglial/macrophage activation in spinal cords of experimental autoimmune encephalomyelitis rats by positron emission tomography using the mitochondrial 18 kDa translocator protein radioligand [^18^F]DPA-714. J Neurosci.

[41] Zinnhardt B, Belloy M, Fricke IB, Orije J, Guglielmetti C, Hermann S (2019). Molecular imaging of immune cell dynamics during de- and remyelination in the cuprizone model of multiple sclerosis by [18F]DPA-714 PET and MRI. Theranostics..

